# Ethnicity, deprivation and screening: survival from breast cancer among screening-eligible women in the West Midlands diagnosed from 1989 to 2011

**DOI:** 10.1038/bjc.2015.204

**Published:** 2015-06-16

**Authors:** M Morris, L M Woods, N Rogers, E O'Sullivan, O Kearins, B Rachet

**Affiliations:** 1 Cancer Research UK Cancer Survival Group, Department of Non-Communicable Disease Epidemiology, Faculty of Epidemiology and Population Health, London School of Hygiene & Tropical Medicine, Keppel St, London WC1E 7HT, UK; 2 West Midlands Breast Screening Quality Assurance Reference Centre, Public Health England, 5 St Philip's Place, Birmingham B3 2PW, UK

**Keywords:** ethnic groups, socio-economic factors, breast neoplasms, survival analysis, mass screening, mammography, Great Britain

## Abstract

**Background:**

Social inequalities in breast cancer survival are smaller when the cancer is screen-detected. We examined survival from screen-detected and non screen-detected breast cancer by ethnicity and deprivation.

**Methods:**

Cancer registry data for 20 283 women aged 50–70 years, diagnosed between 1989–2011 and invited for screening, were linked with screening and ethnicity data. We examined Asian, Black and White groups, less deprived and middle/more deprived women. Net survival was estimated using ethnic- and deprivation-specific life tables. Estimates were corrected for lead-time bias and over-diagnosis.

**Results:**

Net survival varied by screening history. No significant differences in survival were found by ethnicity. Five-year net survival was 90.0% (95% CI, 89.3–90.8%) in less deprived groups and 86.7% (85.9–87.4%) among middle/more deprived women. Screening benefitted all ethnic and both deprivation groups. Whether screen-detected or not, more deprived women had significantly poorer outcomes: 5-year net survival was 78.0% (76.7–79.2%) for deprived women who were not screen-detected compared with 94.0% (93.1–95.1%) for less deprived women who were screen-detected.

**Conclusions:**

The three ethnic groups differed little in their breast cancer survival. Although screening confers a survival benefit to all, there are still wide disparities in survival by deprivation. More needs to be done to determine what underlies these differences and tackle them.

There is evidence that there are substantial socio-economic (Rachet *et al*, 2010) and ethnic (dos Santos Silva *et al*, 2003; Farooq and Coleman, 2005; Jack *et al*, 2009) differences in breast cancer survival: survival is higher in more affluent women, and in those from White and South Asian ethnic groups, and lower among women from more deprived areas and among Black African women.

Possible explanations for these inequalities include later presentation leading to later stage of disease (Sant *et al*, 2003; Woods *et al*, 2006; Jack *et al*, 2009), and differences in screening uptake by both socio-economic status (Maheswaran *et al*, 2006; Gatrell *et al*, 1998) and ethnicity (Szczepura *et al*, 2008; Price *et al*, 2010; Renshaw *et al*, 2010).

Analyses of cancer registry data linked to the National Breast Screening Service records also showed smaller socio-economic and ethnic differences for screen-detected cancer (NHS Cancer Screening Programmes, 2009). However, the overall difference in breast cancer survival between affluent and deprived women was not modified by the introduction of screening in 1989 (Coleman *et al*, 2004).

As ethnic minority women are more likely to be diagnosed with breast cancer at younger ages than White women (NHS Cancer Screening Programmes, 2009; Jack *et al*, 2012), screening has had less potential to reduce the overall ethnic disparities in survival while offered to those over 50. However, as women are invited for screening at a younger age of 47 years, this may change (NHS Cancer Screening Programmes, 2009). Breast cancer incidence also appears to be increasing among ethnic minorities despite historically being lower than that among White groups (Farooq and Coleman, 2005; Jack *et al*, 2009; Stotter *et al*, 2014), which makes this issue even more important.

In this descriptive study, we have used data from a centre of excellence in breast cancer registration (the West Midlands Cancer Registry) to map out the current picture of breast cancer survival among different socio-demographic groups. Our aim was to describe survival differences between ethnic and deprivation groups, and whether screening status has an impact on these. To this end we quantify survival by deprivation, ethnicity and mode of presentation.

## Materials and methods

### Sources of data

The West Midlands Breast Screening Quality Assurance Reference Centre (QARC) has assigned a screening status to every confirmed primary malignant (invasive and *in situ*) breast cancer diagnosed in the region since the start of screening in 1989 (Lawrence *et al*, 2005). This resource is the largest and most complete of its kind in England (UK Association of Cancer Registries, 2010). These data are also linked to the Hospital Episode Statistics (HES) and National Breast Screening Service (NBSS) records, and are regularly linked to the National Mortality Database for updates of vital status. Follow-up was complete up to the end of July 2012 for all women.

### Cohort selection

**Figure 1:**
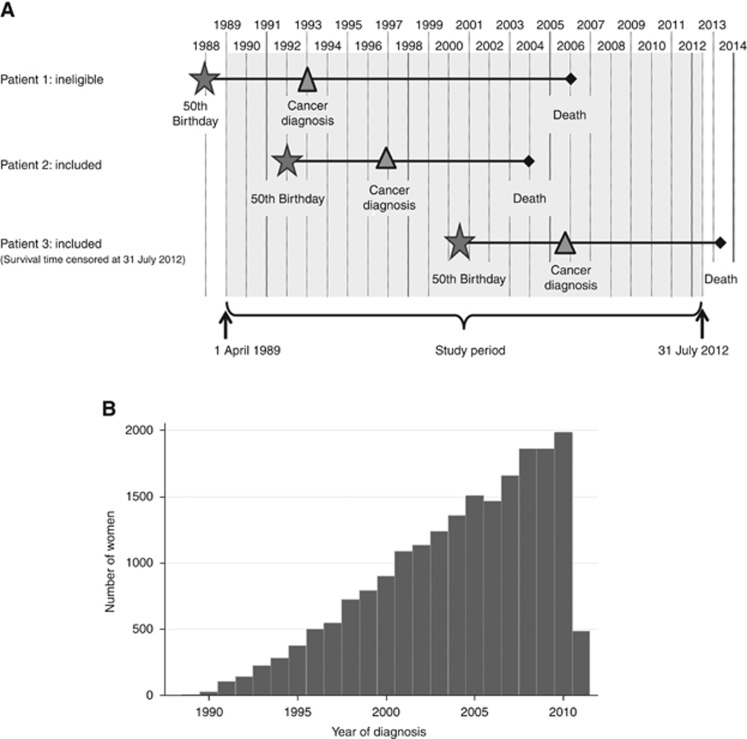
(**A**) Cohort selection diagram and (**B**) accumulation of the cohort through the study period, women diagnosed between 1 April 1989–31 March 2011, followed up to 31 July 2012.

The cohort was defined as women diagnosed with a primary, invasive breast cancer during the period 1 April 1989–31 March 2011 and aged 50–70 years at their diagnosis. It consisted of women who were continuously eligible for screening from the age of 50 up to either 65 or 70 years (if the screening service expanded), who would have received their first invitation letter from their 50th birthday onwards and who would have kept receiving invitation letters until they were a maximum of 70 years old (Figure 1A). Figure 1B shows the accumulation of the cohort over the course of the study period, up to a total of 20 283 women, after excluding 761 women (3.6% of those eligible). These exclusions mainly comprised women whose tumours were recurrences of earlier malignancies.

### Ethnicity data

#### Sources

Data on each woman's ethnicity was gathered from self-reports given on admittance to hospital (from HES data), or in some cases, on presentation for breast screening (from NBSS data). HES records provided ethnicity information for 16 747 women who had, at some point, had an inpatient stay and reported their ethnicity (83% of the cohort). The NBSS provided data for a further 1425 women (7%) where HES data were missing. Among the 6258 observations with information from both sources, the proportion of agreement was 98.6% (Kappa=0.80, *P*<0.001) indicating that the NBSS was a robust source for the missing data.

We imputed the remaining 10% of ethnicity data using name-recognition software, Onomap (Lakha *et al*, 2011; Supplementary Table A). This software uses the first and last names of people in the sample to match to databases of names from different ethnicities, and so can be used to impute ethnicity where no other information exists.

#### Categorisation

Some women had several HES records due to repeated inpatient stays, and some had multiple ethnicities reported. A hierarchy was applied in order to derive a single code for these women as follows: (i) the most common code was used, (ii) the most recent code was used, if there was an equal number of one code and (iii) alphanumerically, if there was an equal number of one code on same date (∼0.001% patients). Three main ethnic categories were created: Asian (all groups including from mixed heritage), Black (all groups including from mixed heritage) and White (including mixed heritage other than Black/Asian and ‘other' Supplementary Table A).

### Assigning deprivation score

Two different ecological measures of deprivation were used according to the study period: an updated Townsend score (Townsend *et al*, 1988) for women diagnosed with a breast cancer between 1990 and 2000 (40% of cases); the 2004, 2007 and 2010 scores of the Income Domain of the Index of Multiple Deprivation (IMD; 21.4%, 26.5% and 12.2%, respectively) for women diagnosed from 2001 onwards. The women were assigned a score according to their geographical area of residence at the time of their diagnosis (Department of the Environment Transport and the Regions, 2000; Neighbourhood Renewal Unit, 2004). The IMD scores used Lower Super Output Areas (∼1500 people), whereas electoral wards had been used previously (average population of 5500 people). The scores were split into five categories based on the quintiles of the national distribution of the areas.

### Screening history

Breast cancer diagnoses in the cancer registry are linked to the woman's screening history by the QARC. Women were categorised according to how their cancer was diagnosed and their screening history: (i) women with screen-detected cancer (no matter how many previous screens she may have had), (ii) women whose last screening attendance had resulted in a negative screen and had not yet been invited to a subsequent screening (interval cancer), (iii) those whose cancer was diagnosed after having previously had a negative screen, but who had not attended their most recent screening appointment (lapsed attenders), (iv) women who had never presented for screening (non-attenders). The last three categories encompass women whose cancer has been detected symptomatically and were grouped for the main analyses as not screen-detected.

### Stage of disease

The West Midlands Cancer Registry provided information on tumour size, node involvement and presence of metastases. This information was converted into a variable for ‘extent of disease' (localised=confined to the organ of origin, regional=spread to adjacent muscle, organ, fat, connective tissue or regional lymph nodes, and distant=distant metastases).

### Analysis

We estimated net survival using the non-parametric Pohar Perme estimator (Pohar Perme *et al*, 2012) implemented in *stns:* software available for Stata 13 (StataCorp, 2013). Net survival provides an estimate of survival from the cancer itself, adjusting for expected mortality from other causes. The Pohar Perme estimator has been shown to provide unbiased estimates of net survival from population-based data (Danieli *et al*, 2012; Roche *et al*, 2013).

We estimated the expected survival from ethnic life tables for England and Wales. We have constructed ethnic-majority life tables for 2001 (Morris *et al*, 2015) making use of census data about the ethnic make-up of very small geographical areas, and mortality data provided by the Office for National Statistics. We interpolated life tables up to 2012 on the bases of these 2001 ethnic-specific tables and the England and Wales life tables for each of the 2002–2012 years.

To account for the potential effect of lead-time bias in the screen-detected group, we applied the method established by Duffy *et al* (2008). For each patient, additional survival time due to screening, *E(s)*, was estimated, assuming an exponential distribution of survival times and mean sojourn time of 4 years (Duffy *et al*, 2008). In order to account for the uncertainty associated with this value, we then generated ten separate data sets for the screen-detected group containing *E(s)*_*1*_, *E(s)*_*2*_ … *E(s)*_*10*_ assuming these values were exponentially distributed with a mean of *E(s)*. This resulted in a range of estimates for the possible additional survival due to lead time which were then subtracted from observed survival time in order to obtain corrected survival time.

We considered tumours to be over-diagnosed if they would not have been detected symptomatically during the study period or during the woman's predicted life time. We therefore excluded tumours in instances where the value of *E(s)* exceeded the woman's actual observed survival time, either because the predicted date of diagnosis was after 31 March 2011, or in excess of her life expectation at diagnosis.

We used the corrected survival times to estimate non-parametric net survival for each data set of the screen-detected group. We applied the rules established by Rubin (1987) for the re-combination of estimates in a multiple-imputation setting to derive an overall estimate of net survival and its variance, adjusted for lead-time bias and over-diagnosis.

We applied locally weighted regression to smooth the survival estimates (Cleveland, 1979; Royston, 1991), using a conservative degree of smoothing to maintain the variability evident in the more sparse data. When corrected estimates were used (in comparisons involving screen-detected women), however, smoothing was not applied as there were too few data points in some of the groups for the patterns to be evident.

## Results

### Cohort characteristics

The distribution of women by deprivation varied substantially by ethnicity. White women were more likely to be affluent than either Asian or Black women (Table 1). The mean age of women in the cohort was 57.5 years (s.d.=5.0 years). The White group of women were somewhat older.

The proportion of women diagnosed with localised disease was 60.3% in the White group, but much lower among Asian women (55.8%) and particularly among Black women (46.6%). Black women had a commensurately higher proportion of regional disease (45.5% compared with 35.8% in Asian women and 31.1% in White women, *P*<0.001).

Half of the White (51.9%) and Asian (50.0%) women in the study had screen-detected breast cancer, but among Black women the proportion was lower (45.0%, *P*<0.001) (Table 2). Although Asian women had the smallest proportion of interval cancers (24.2%), they were the most likely to be lapsed or non-attenders (*P*<0.001). Screening status also varied by deprivation: there was little difference in the proportions that were screen-detected across the deprivation categories, but there was a clear increase in the proportion who were lapsed or non-attenders from the least to the most deprived (*P*<0.001).

### Survival analysis

#### Survival by ethnicity

**Figure 2:**
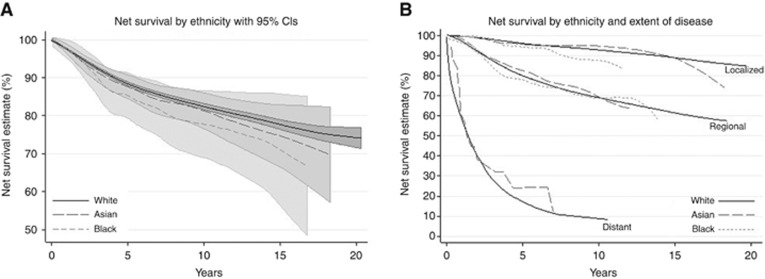
Net survival (**A**) by ethnicity, showing 95% confidence intervals (CIs), and (**B**) by ethnicity and extent of disease, corrected for background mortality using ethnic life tables adjusted for deprivation. Note that for clarity, CIs are not shown on graphs from now on where they fully overlap; too few data points in the Black group for distant disease (graph B) to show after smoothing.

Figure 2A shows net survival from breast cancer for each ethnic group. Women in the Black groups show consistently lower survival than the other groups, but there are no significant differences found, because the confidence intervals are wide for the minority groups and they overlap fully at every time point.

All ethnic groups had high survival when the disease was localised at diagnosis. Survival was much lower among those with regional and distant disease. There was very little survival difference between the ethnic groups, except some slight suggestion that Black women fared worse for long-term survival than other groups when their disease was localised (Figure 2B).

Survival has improved in each time period overall and for Asian and White ethnic groups, but no significant gains could be seen in Black groups (Supplementary Figure A). However, there were too few data points in the earliest time period in the Asian and Black groups to show after smoothing.

#### Survival by deprivation

**Figure 3:**
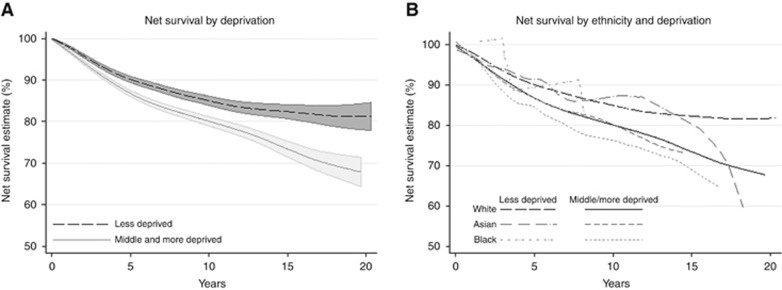
Net survival by deprivation (**A**), and by ethnicity and deprivation (**B**). Note that for clarity, CIs are not shown on graphs where they fully overlap.

There was a significant survival difference between the less deprived and the middle and more deprived groups (Figure 3A) at every follow-up time. Five-year survival, for example, was 90.0% (95% CI, 89.3–90.8%) in less deprived groups and 86.7% (85.9–87.4%) in the middle and more deprived groups, with gaps widening with time.

Survival was similar for each ethnicity when split by deprivation, however (Figure 3B). Survival for the middle and more deprived Black women seemed to be lower than in similarly deprived women in the other two ethnic groups. There were very few Black women in the higher socio-economic categories, which made a robust comparison difficult.

There was a significant survival difference for White women between the less deprived *vs* the middle and more deprived groups, similar to that seen in the overall graph (Figure 3A). Small numbers in the two minority ethnic groups prohibited a valid comparison by deprivation (data not shown).

#### Survival by screening status

In all ethnic and deprivation groups, those with screen-detected cancer had the best 5-year survival, whereas non-attenders had the poorest, after those diagnosed with interval cancers and lapsed attenders (Table 2).

The graphs below use estimates for screen-detected women corrected for lead-time bias and potential over-diagnosis so that their results can be compared, without bias, with those of non screen-detected women.

**Figure 4:**
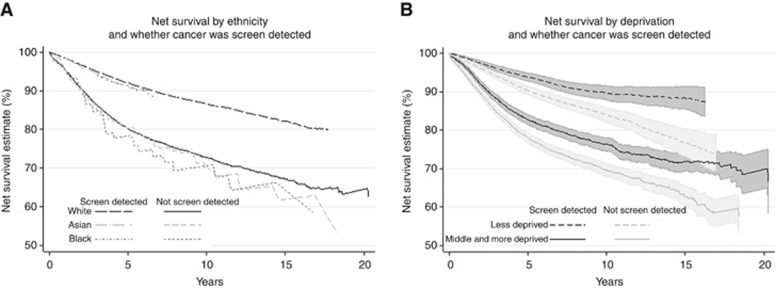
Net survival according to screening history, by ethnicity (**A**) and deprivation (**B**). Note that estimates for the screen-detected groups shown here have been adjusted for lead-time bias and over-diagnosis. For clarity, CIs are not shown on graphs where they fully overlap.

There was a clear benefit in all ethnic groups for those who have been screen-detected *vs* those who have not (Figure 4A) and there were no evident ethnic differences within screening categories. Survival was more similar for the screen-detected group, whilst Black women appeared to have consistently lower survival in the non screen-detected group. However, these differences were not statistically significant.

In contrast, clear differences were found between deprivation groups within each category of screen detection. Screen detection again conferred a clear benefit in both deprivation groups but, whether screen-detected or not, women in the middle and more deprived categories had significantly poorer survival (Figure 4B). At 5 years, there was a difference of 16% between the net survival of highest surviving group (less deprived women who were screen-detected, 94.0% 95% CI, 93.1–95.1%) and the lowest surviving group (the middle and more deprived women who were not screen-detected, 78.0% 95% CI, 76.7–79.2%). The gap was even wider at 10 years.

## Discussion

This study describes the survival from breast cancer of a cohort of women from the West Midlands, by their ethnicity, period of diagnosis, extent of disease at diagnosis, deprivation status and screening history. After correcting for background mortality for each group, survival from breast cancer was not found to vary significantly by ethnic group in this sample. There was also little difference between ethnicities when examining survival by extent of disease or by deprivation. However, a deprivation gap was apparent overall. We found clear evidence of a survival benefit of screening for women in all ethnic groups, and in different deprivation groups. However, large differences were also found within screening categories for the deprivation groups, with those less deprived showing a clear benefit whether screen-detected or not.

Women who were diagnosed in the earliest time periods had lower survival. However, excluding these women did not impact the inequalities seen by deprivation. In the most recent time periods, there is little evidence of a deprivation gap up to 5 years after diagnosis (data not shown), but given that the deprivation gap widens beyond 5-year survival in earlier time periods, it remains to be seen whether the discrepancy will still be evident with longer follow-up in those diagnosed more recently.

We have been able to compare patterns of survival from breast cancer between the three main ethnic groups among screening age women for the first time. Because of small numbers in minority groups, we used broad groupings of ethnicity. We also added women self-identified as ‘mixed ethnicity' into the minority group that they identified as part of their mixed background. This decision was based on the fact that the ‘mixed' category is heterogeneous partly because of the different minorities represented (Bradford, 2006; Platt, 2011). However, there is little literature confirming that these women are more likely to be more similar to their minority background than to White groups in their health behaviours.

We will have missed some of the diversity in health outcomes still to be found within these broad ethnic groups (Atkin *et al*, 2010). These broad categories tell us little regarding the cultural or faith differences within them. These might themselves have a large impact on healthcare-seeking behaviour, lifestyle choices and support structures (Acheson, 1998; Renshaw *et al*, 2010). Expanding this study to cover the whole of UK would allow us to examine some more fine-grained differences.

Despite being limited to the West Midlands area, the data came from a centre of excellence for breast cancer registration. They contained only women who would have been invited for screening, and were linked with several data sources to achieve highly complete self-reported ethnic identity, augmented by imputation from names for only 10% of the cohort. The completeness of self-reported ethnicity information is much higher than in many studies which have attempted to quantify the effect of ethnicity on breast cancer survival: completeness was generally around two thirds (NHS Cancer Screening Programmes, 2009; Jack *et al*, 2009; 2012) or three quarters (National Cancer Intelligence Network and Cancer Research UK, 2009). Despite recent improvements, the Second All Breast Cancer Report still achieved lower completeness (80%) in their 2007 data for England, and Davies *et al* (2013) achieved 77% in London.

This method of linking different sources of ethnicity data has been used successfully elsewhere, and is advocated for improving ethnicity data (Aspinall and Jacobson, 2007). Onomap sensitivity is high for White and Asian names (99.8% and 82.1%, respectively), but very low for Black names (4.4% Ryan *et al*, 2012). We compared Onomap-assigned ethnicity in our sample with self-reported ethnicity for those women with both sources of ethnicity information. This gave a sensitivity of 99.6%, 80.8% and 3.3% for White, Asian and Black names, respectively, and specificities of 14.9%, 99.5% and 99.9%, respectively, which corresponds closely with that previously found.

Where women had multiple ethnicities reported, which could be due to changing self-perception, recording errors or changes in coding methods, the algorithm used by the West Midlands Breast Screening QARC ensured a consistent approach for dealing with them.

### Appropriate adjustments and corrections

It is essential in net survival analysis to use life tables which account as closely as possible for the background mortality specific to each sub-group (Dickman *et al*, 1998; Blakely *et al*, 2012). The recent derivation of deprivation-adjusted ethnic life tables (Morris *et al*, 2015) enables us to apply the best possible correction (Supplementary Figure B). When we compared the net survival estimates after correcting with these life tables with those corrected only with regional, deprivation-specific life tables, we saw smaller changes (not shown). For instance, breast cancer survival in Black women was slightly lower using the regional life tables. This means that differences in survival between ethnicities appear greater if ethnic life tables are not used.

A concern when estimating survival has been the lead-time effect (Hutchison and Shapiro, 1968) in which survival time appears artificially increased as tumours are diagnosed earlier by screening than they would have been symptomatically, but without improvement in prognosis (Ellis *et al*, 2014). The correction we applied, however, enables a direct comparison between women with screen-detected and non screen-detected cancers (Duffy *et al*, 2008; Lawrence *et al*, 2009). In addition we corrected for potential over-diagnosis, ensuring that those women whose cancer would not have been diagnosed symptomatically during the study period or during the patient's life time were excluded. We applied these corrections in any comparison of screen-detected and non screen-detected women to enable an unbiased comparison. Omitting these corrections would have over-estimated long-term net survival for screen-detected women (Supplementary Figure C).

Length bias can also exaggerate the potential survival advantage of screen-detected women in comparison with non screen-detected women. This is the tendency for slower growing tumours with better overall prognosis to be more likely to be detected by mammography. Duffy *et al* (2008) have shown, by means of a sensitivity analysis, that the impact of this bias is relatively small, reducing the 10-year survival for the screen-detected group by only 1%.

### Comparison with other studies

There are some discrepancies in results between studies. Some have found slightly higher survival from breast cancer for South Asian women, for example, compared with non-South Asians (dos Santos Silva *et al*, 2003; Farooq and Coleman, 2005). These studies, which assigned ethnicity with a name-based software (SANGRA (Nanchahal *et al*, 2001)), used national life tables to account for the background mortality. By contrast, the NHS All Breast Cancer reports, based on 2006 and 2007 data, found a small, non-significant, survival advantage for Asian women but the 5-year relative survival for Black and White groups were found to be the same as each other (NHS Cancer Screening Programmes, 2009; National Cancer Intelligence Network, 2011). These studies used deprivation-specific national life tables. Our study corrected for background mortality by both deprivation and ethnicity. These corrections are crucial because the proportion of women in our data from more deprived categories was much higher in the Asian and Black ethnic groups, compared with that in the White groups. Similar proportions were found in the NHS All Breast Cancer Reports (NHS Cancer Screening Programmes, 2009; National Cancer Intelligence Network, 2011) for White groups, using data from all of England using 2001 Census information.

The benefit of screening to all groups tallies with findings from several other studies (Lawrence *et al*, 2009; NHS Cancer Screening Programmes, 2009; National Cancer Intelligence Network, 2011), as does the deprivation gap seen (NHS Cancer Screening Programmes, 2009; Rachet *et al*, 2010; National Cancer Intelligence Network, 2011; Davies *et al*, 2013). The same caveat applies as above, however, as this study takes these results a step further by using the most up-to-date methods of net survival analysis and, for the first time, the ethnic- and deprivation-specific life tables.

Half of our cohort had screen-detected breast cancer which contrasts to some other studies which found lower proportions, especially among Black women (NHS Cancer Screening Programmes, 2009; National Cancer Intelligence Network, 2011; Davies *et al*, 2013), and also in Indian women (Davies *et al*, 2013). This could be due to the fact that we restricted our sample only to women who would have been invited for screening, rather than just identifying all women within the screening age range, as is commonly done.

The Black women in our sample were slightly less likely to have been screen-detected than the other ethnic groups and the Asian women were more likely than the White group to be lapsed or non-attenders. The age range chosen for our sample might explain some differences to other studies, as breast cancer is more likely to occur before age 50 in Black women (NHS Cancer Screening Programmes, 2009; National Cancer Intelligence Network, 2011). This also explains why in this sample the White women were older than the women in other groups. It may also be related to lower screening uptake among more deprived socio-economic groups (Banks *et al*, 2002; Maheswaran *et al*, 2006; NHS Cancer Screening Programmes, 2009) as a greater proportion of Black women reside in these areas. Some researchers contend, however, that socio-demographic factors are not solely responsible for uptake, but that health behaviour differences and other experiences with cancer may be more important (Lagerlund *et al*, 2000; Szczepura *et al*, 2008).

Uptake of screening among ethnic groups is likely to have changed over the time covered by our data. Some research, which adjusted for deprivation and age, has found that although there may still be ethnic differences in uptake, these may be decreasing as time goes on (Szczepura *et al*, 2008). The similarity or difference in uptake by different ethnic groups would not have directly impacted our results as we have examined survival among those whose cancer was screen-detected and among those whose cancer was not.

## Conclusions

This study showed a heartening lack of difference between ethnic groups in survival from breast cancer over the course of time since screening started, although the deprivation gap was still very much apparent despite screening attendance. By using the best analysis methods to describe the data and correcting for potential biases, we have produced a robust result that can now be built upon. The overriding message is that, for all ethnic groups, attendance at screening has a beneficial long-term effect on survival, however more work needs to be done to tackle economic inequalities in survival. Initiatives to inform women about breast cancer screening programmes, and so enable them to make an informed choice regarding attendance, should prioritise women from the more deprived communities.

Future research will explore various potential explanatory factors through multivariable modelling, including the timing of treatments received and of tumour biology, as these could go some way to explain the survival differences found between deprivation groups. Larger studies that use UK-wide data would be able to investigate patterns hidden within these broader ethnic groups.

## Figures and Tables

**Table 1:** Cohort characteristics by ethnicity

***n*****=20 283**	**Asian,** ***n*** **(%)**	**Black,** ***n*** **(%)**	**White,** ***n*** **(%)**	**Total,** ***n*** **(%)**	***P*****-value (*****χ***^**2**^)^a^
**Age group at diagnosis (**years)					
50–54	230 (39.0)	76 (39.8)	6575 (33.7)	6881 (33.9)	0.006
55–59	189 (32.0)	60 (31.4)	6096 (31.3)	6345 (31.3)	
60–64	128 (21.7)	34 (17.8)	4703 (24.1)	4865 (24.0)	
65–70	43 (7.3)	21 (11.0)	2128 (10.9)	2192 (10.8)	
**Period of diagnosis**					
1989–1994	21 (3.6)	8 (4.2)	772 (4.0)	801 (3.9)	0.077
1995–2000	92 (15.6)	30 (15.7)	3719 (19.1)	3841 (18.9)	
2001–2006	214 (36.3)	76 (39.8)	7502 (38.5)	7792 (38.4)	
2007–2011	263 (44.6)	77 (40.3)	7509 (38.5)	7849 (38.7)	
**Vital status at the end of follow up**					
Dead	103 (17.5)	43 (22.5)	3586 (18.4)	3732 (18.4)	0.286
**Deprivation quintile**					
Least deprived	48 (8.1)	10 (5.2)	4420 (22.7)	4478 (22.1)	<0.001
2	52 (8.8)	20 (10.5)	4542 (23.3)	4614 (22.7)	
3	86 (14.6)	25 (13.1)	4065 (20.8)	4176 (20.6)	
4	113 (19.2)	33 (17.3)	3400 (17.4)	3546 (17.5)	
Most deprived	291 (49.3)	103 (53.9)	3057 (15.7)	3451 (17)	
Missing	0 (0)	0 (0)	18 (0.1)	18 (0.1)	
**Deprivation group**					
Less deprived (quintiles 1+2)	100 (16.9)	30 (15.7)	8962 (46.0)	9092 (44.8)	<0.001
Middle and more deprived (quintiles 3+4+5)	490 (83.1)	161 (84.3)	10 522 (54.0)	11 173 (55.1)	
Missing	0 (0)	0 (0)	18 (0.1)	18 (0.1)	
**Extent of disease**					
Localised	329 (55.8)	89 (46.6)	11 758 (60.3)	12 176 (60.0)	<0.001
Regional	211 (35.8)	87 (45.5)	6066 (31.1)	6364 (31.4)	
Distant	18 (3.1)	3 (1.6)	462 (2.4)	483 (2.4)	
Missing	32 (5.4)	12 (6.3)	1216 (6.2)	1260 (6.2)	
Total	590 (100.0)	191 (100.0)	19 502 (100.0)	20 283 (100.0)	

a *χ*^2^ test performed on non-missing data only.

**Table 2:** Net survival for women with different screening histories, by ethnicity and deprivation

	**Total**	**Screen-detected**	**Interval**	**Lapsed attender**	**Non-attender+other**^a^
**Total**	**20 283 (100)**	**10 509 (51.8)**	**6313 (31.1)**	**1143 (5.6)**	**2318 (11.4)**
**Ethnicity**^b^					
Asian *n* (%)	590 (100)	295 (50.0)	143 (24.2)	48 (8.1)	104 (17.6)
Net survival	1 year	98.5% (96.6–100.3%)	97.5% (94.8–100.2%)	92.1% (84.3–99.8%)	93.5% (88.7–98.4%)
	5 years	91.3% (86.7–96.2%)	83.8% (76.8–90.8%)	82.3% (70.4–94.2%)	75.5% (66.3–84.7%)
Black *n* (%)	191 (100)	86 (45.0)	66 (34.6)	11 (5.8)	28 (14.7)
Net survival	1 year	—	98.7% (95.8–101.6%)	91.0% (74.8–107.2%)	89.8% (78.4–101.1%)
	5 years	92.0% (91.2–92.8%)	79.2% (68.0–90.37%)	—	71.8% (53.8–89.8%)
White *n* (%)	19 502 (100)	10 128 (51.9)	6104 (31.3)	1084 (5.6)	2186 (11.2)
Net survival	1 year	98.4% (98.1–98.7%)	97.6% (97.2–98.0%)	95.4% (94.1–96.7%)	88.9% (87.6–90.3%)
	5 years	91.2% (82.4–101.0%)	84.3% (83.3–85.4%)	78.9% (76.0–81.9%)	68.9% (66.7–71.0%)
**Deprivation quintile**^b^					
Least deprived	4478 (100)	2320 (51.8)	1562 (34.9)	217 (4.8)	379 (8.5)
Net survival	1 year	98.9% (98.3–99.4%)	98.1% (97.3–98.9%)	96.7% (94.2–99.2%)	90.1% (87.0–93.1%)
	5 years	93.9% (92.5–95.4%)	86.1% (84.1–88.1%)	81.2% (75.0–87.3%)	73.0% (68.1–77.9%)
2	4614 (100)	2380 (51.6)	1534 (33.2)	251 (5.4)	449 (9.7)
Net survival	1 year	98.8% (98.2–99.4%)	98.1% (97.4–98.9%)	95.0% (92.1–97.8%)	90.8% (88.1–93.5%)
	5 years	93.7% (92.2–95.3%)	84.4% (82.3–86.5%)	81.41 (75.7–87.1%)	74.3% (69.9–78.8%)
3	4176 (100)	2232 (53.4)	1296 (31.0)	219 (5.2)	429 (10.3)
Net survival	1 year	98.5% (97.8–99.2%)	97.7% (96.8–98.6%)	94.6% (91.5–97.8%)	89.4% (86.5–92.4%)
	5 years	91.9% (90.2–93.7%)	84.7% (82.4–87.0%)	80.91% (74.6–87.2%)	69.3% (64.5–74.2%)
4	3546 (100)	1830 (51.6)	1032 (29.1)	193 (5.4)	491 (13.8)
Net survival	1 year	98.1% (97.4–98.9%)	97.4% (96.3–98.4%)	94.8% (91.6–98.1%)	87.9% (85.0–90.9%)
	5 years	89.8% (87.7–91.9%)	81.5% (78.7–84.2%)	74.45 (66.9–82.0%)	69.0% (64.5–73.4%)
Most deprived	3451 (100)	1740 (50.4)	887 (25.7)	262 (7.6)	562 (16.3)
Net survival	1 year	97.6% (96.7–98.5%)	96.7% (94.2–99.2%)	94.8% (92.0–97.7%)	87.9% (85.2–90.7%)
	5 years	89.0% (86.8–91.1%)	81.2% (75.0–87.3%)	76.71 (70.5–83.0%)	62.3% (57.9–66.8%)
Missing data	18 (100)	7 (38.9)	2 (11.1)	1 (5.6)	8 (44.4)

Note: Confidence intervals over 100% indicate that the survival of the group is not significantly different from the background mortality found in the life tables.

a Ceased/not known/irresolvable *n*=276 (1.4%).

b *χ*^2^ test comparing proportion of each ethnic group by screening history *P*<0.001; comparing each deprivation group by screening history *P*<0.001.
