# Supplementary material for: Ethnicity, deprivation and screening: survival from breast cancer among screening-eligible women in the West Midlands diagnosed from 1989 to 2011
Source: Br J Cancer. 2015 Jun 16;113(3):548–55. doi: 10.1038/bjc.2015.204 (PMC4522622; doi:10.1038/bjc.2015.204)
Supplement: Supplementary Information [file bjc2015204x1.doc]

**Web appendix**

Table A: Distribution of ethnicity data in sample showing sources and imputation

|  | **Self-reported ethnicity from HES + NBSS*** | | **Distribution of missing**  **data by Onomap** | | **Final distribution:**  **HES + NBSS with**  **Onomap imputation** | |
| --- | --- | --- | --- | --- | --- | --- |
| **Ethnicity** | **n** | **%** | **n** | % | **n** | **%** |
| White + other | 17,441 | 86.0 | 2,056 | 97.4 | 19,497 | 96.1 |
| Asian | 543 | 2.7 | 47 | 2.2 | 590 | 2.9 |
| Black | 188 | 0.9 | 3 | 0.1 | 191 | 0.9 |
| *Missing* | *2,111* | *10.4* | *5* | *0.2* | *5* | *0.0* |
| **Total** | **20,283** | **100.0** | **2,111** | **100.0** | **20,283** | **100.0** |

*HES = Hospital Episode Statistics, NBSS = National Breast Screening Service

Figure A: Net survival by ethnicity and period of diagnosis, corrected for background mortality using ethnic life tables adjusted for deprivation, showing 95% confidence intervals (CIs).

(Note: For clarity, CIs not shown on graphs where they fully or mostly overlap)

**b**

**a**

Figure B: Comparison of survival estimates: (a) Kaplan-Meier estimate: not corrected for background mortality; (b) Net survival (unsmoothed): corrected for background mortality using ethnic life tables adjusted for deprivation.

Figure C: Comparison of net survival among screen-detected women, corrected and not corrected for lead time bias and overdiagnosis
